# Spontaneous rupture of a large splenic artery aneurysm in a 59-year-old male patient with pemphigus vulgaris: a case report

**DOI:** 10.1186/s13256-022-03618-x

**Published:** 2022-10-21

**Authors:** Ahmad Hosseinzadeh, Reza Shahriarirad, Vahid Asgharzadeh Majdazar, Mohammad Moeini Farsani, Seyed Mohammad Kazem Tadayon

**Affiliations:** 1 Thoracic and Vascular Surgery Research Center, Shiraz University of Medical Science, Shiraz, Iran; 2 School of medicine, Shiraz University of Medical Science, Shiraz, Iran; 3 Department of Surgery, Shiraz University of Medical Sciences, Shiraz, Iran; 4 Colorectal Research Center, Shiraz University of Medical Sciences, Shiraz, Iran

**Keywords:** Splenectomy, Aneurysm, Ruptured, Splenic artery, Vascular, Artery, Pemphigus vulgaris

## Abstract

**Background:**

There is currently no information on the anatomical risk factors for splenic artery aneurysm rupture, specifically the location or size of the lesion; therefore, reporting this entity to obtain data and ultimately reduce morbidity and mortality is essential. Here we report a case of a male patient with spontaneous rupture of a large splenic artery aneurysm presenting with abdominal pain.

**Case presentation:**

A 59-year middle-eastern male, with known pemphigus vulgaris presented with a chief complaint of headache and syncope, followed by abdominal pain along with severe metabolic acidosis. A contrast-enhanced computed tomography scan of the abdomen and pelvic showed a splenic artery aneurysm of 33 × 30 mm with a 150 × 90 mm hematoma formation around the aneurysm site. The patient underwent an operation and splenectomy, with confirmation of the diagnosis of ruptured splenic artery aneurysm.

**Conclusion:**

It is essential to consider splenic aneurysm rupture as a second-line differential diagnosis, especially among patients with comorbid diseases, as this can lead to timely and appropriate lifesaving intervention.

## Introduction

Splenic artery aneurysms (SAA) are the third most common type of arterial aneurysm, with diameters ranging from 0.6 to 30 cm. The majority of patients show no signs or symptoms [1]. The exact cause of a splenic artery aneurysm is uncertain, while its combination with the possibility of rupture, can result in a clinical picture ranging from nonspecific abdominal symptoms (making prerupture diagnosis difficult) to a more dramatic intraperitoneal hemorrhage with hypovolemic shock and high morbidity and mortality, making it a retrospective diagnosis [2]. There is currently no information on the anatomical risk factors for SAA rupture, especially the location or size of the lesion; therefore, reporting this entity to obtain data for decreasing mortality and morbidity is essential. Here we report a male patient with the cutaneous disease of pemphigus vulgaris, who developed a spontaneous rupture of a large SAA, presenting with abdominal pain.

## Case report

A 59-year middle-eastern male, with known pemphigus vulgaris treated with a daily dose of 5 mg prednisolone, presented to the emergency department with a chief complaint of headache and syncope 3 days before presentation, with subsequent onset of severe abdominal pain. On admission, the patient’s Glasgow Coma Scale level was 13, he was tachycardic (rate: 130) and had severe epigastric pain, along with left upper quadrant pain and tenderness, without radiation or association with nausea and vomiting. Arterial blood gas evaluation demonstrated severe metabolic acidosis. Electrocardiography only showed premature ventricular complexes. Abdominal pelvic sonography demonstrated free fluid in the upper pole of the left kidney and around the spleen, without stasis.

Initial laboratory data demonstrated white blood cell levels of 18.8 (neutrophil 81.4% and lymphocyte of 9.2%), hemoglobin of 7.7 g/dL, platelet count of 179 × 10^3^/μL, creatinine of 1.9 µmol/L, and blood urease nitrate (BUN) of 30 mmol/L. Initial arterial blood gas had a pH of 7.19 with PaCO_2_ of 44.4 mmHg, PaO_2_ of 22.6 mmHg, HCO_3_ of 16.6 mmol/L, and base excess of −11.1, which then improved to PpH 7.36, PaCO_2_ 31.7 mmHg, PaO_2_ 57 mmHg, HCO_3_ 17.7 mmol/L, and base excess −6.4 after fluid resuscitation.

**Fig. 1:**
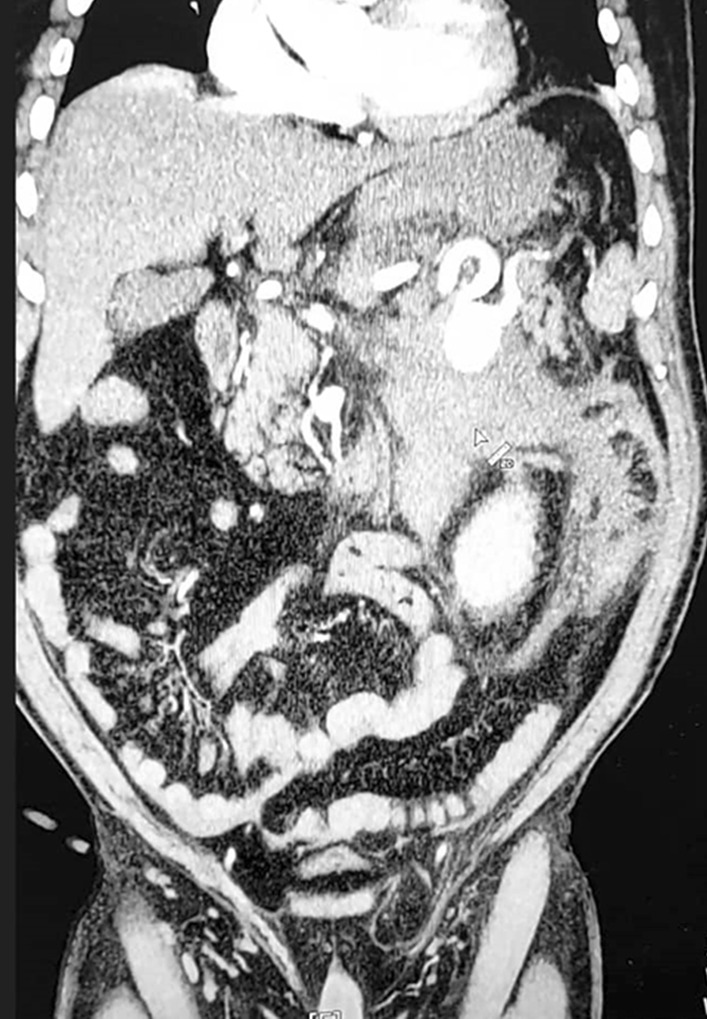
Computed tomography scan of a 59-year-old man with a ruptured splenic aneurysm

Brain computed tomography (CT) and echocardiography were requested for the patient, which were unremarkable. A contrast-enhanced computed tomography scan of the abdomen and pelvis showed a splenic artery aneurysm of 33 × 30 mm with 150 × 90 mm hematoma formation around the aneurysm site (Fig. 1). Based on the patient’s condition, he was immediately transferred to the operating room, where a diagnosis of ruptured splenic artery aneurysm was made.

**Fig. 2:**
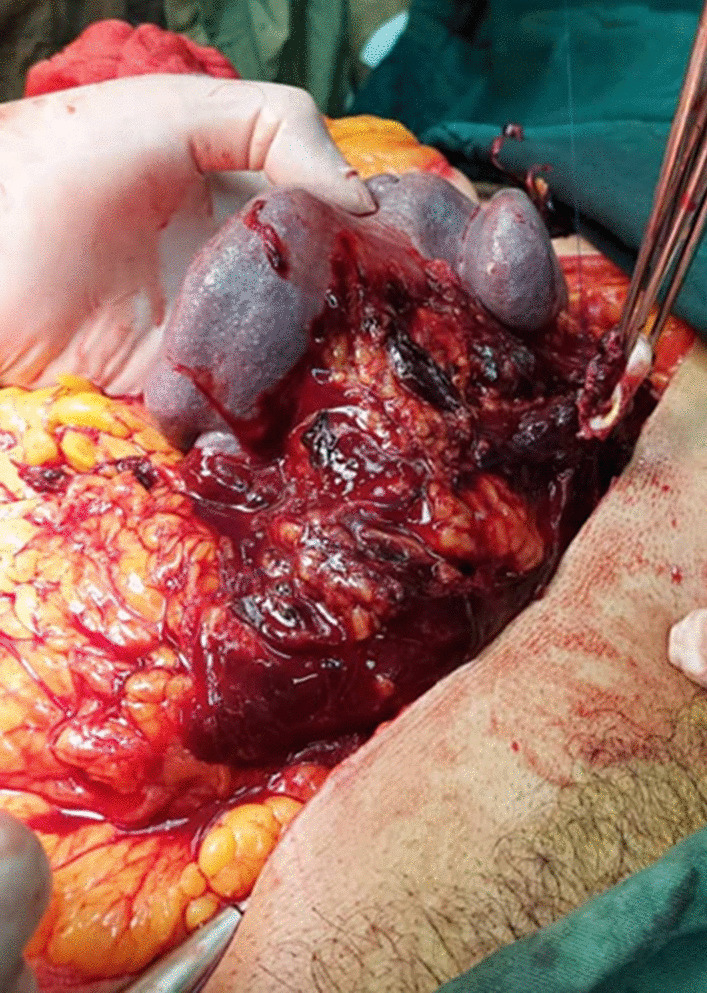
Ruptured aneurysm with hematoma

An exploratory laparotomy was performed, and a 1200 cc blood clot in the abdominal cavity was observed, along with a massive retroperitoneal hematoma that extended to the pelvic cavity, and a hematoma at the meso of the transverse colon. Initially, the lesser sac was explored to gain proximal and distal control, but owing to lack of access to the aneurysm, which was at the middle part of the splenic artery, left medial visceral rotation was performed. The spleen was mobilized, an aneurysm sac was detected and ligated, and a splenectomy was performed (Fig. 2). A Nelaton drain was also inserted.

The patient had no postoperative complications: the drain was removed on the 7th day, and thrombocytosis was treated with aspirin. Pneumovax was injected in the following days. During his 1-week follow-up, he had achieved full recovery and had no sign of any complications and normal laboratory data. He was under routine follow-up and steroids were tapered and discontinued. He also had no complications or cutaneous manifestations.

## Discussion

Ruptured SAAs are an uncommon cause of hemorrhagic shock, but the splenic artery accounts for 60% of visceral aneurysms [3]. SAA has been most frequently reported in females, with a 4:1 female-to-male ratio [3–5], although rupture may occur more frequently in men [6]. The majority of SAAs (> 90%) are asymptomatic and are discovered incidentally during imaging studies [7]. SAAs are also more common after the sixth decade of life, with 80% of cases occurring in patients over 50 years of age [1], similar to our case. In contrast to pseudoaneurysms, typically associated with pancreatitis and iatrogenic instrumentation, true SAA retains all three layers of the blood vessel but is thinned out.

Patients are often asymptomatic, and when symptoms are present, they are often vague and varied (17%) [4], making a prerupture diagnosis challenging, especially when the aneurysm is tiny. When an aneurysm ruptures, it can cause a dramatic hypotensive shock with a high mortality rate, typically resulting in death outside of the hospital or a rare double-rupture occurrence. The interval between these two episodes can be as brief as a few hours to many days [8–10], providing a window for lifesaving action. If SAA rupture is still not recognized in the diagnosis, this can cause confusion in the clinical picture. The rupture occurs in nearby organs such as the stomach, pancreas, or colon in 30% of cases [11, 12]. SAAs generate considerable blood loss after rupture, with hemodynamic instability appearing in 6–96 hours, affording time for repair if detected. Non-pregnant patients have a mortality rate of 10–36%. [13, 14]; however, for pregnant women and those with pre-existing portal hypertension, this number doubles [14]. Wiener *et al.* [15] proposed an approach regarding the management of SAA in pregnancy, for which, in asymptomatic cases with an SAA of less than 2 cm, conservative surveillance can be applied. Planned cesarean delivery at 34–36 weeks gestation followed by elective SAA embolization will evade extreme premature delivery as well as splenectomy and radiation hazard during pregnancy. However, since SAA has been associated with high mortality and rapid progression in pregnancy [16], it is vital to achieve diagnosis and treatment as soon as possible.

The condition is largely diagnosed on contrast CT performed for abdominal pain, and is seen as free fluid around the spleen or other viscera and as leaked contrast media or contrast-enhanced hematoma. As part of the workup for abdominal pain, a ruptured SAA with intra-abdominal hemorrhage may be discovered. Free fluid around the spleen and other viscera is usually detected by CT, and contrast media leakage or contrast-enhanced hematoma may also be present. Because the majority of aneurysms develop late in pregnancy, placental abruption is the most common misdiagnosis, and it is usually only corrected during exploration, and complex diagnostic facilities are not readily available. Due to the double rupture phenomenon, aneurysm rupture can be difficult to diagnose at first. Rupture into the lesser omental can temporarily tamponade bleeding and cause acute abdominal pain and hypotension. As the bleeding continues, the lesser sac ruptures, releasing the tamponade and resulting in massive abdominal bleeding and cardiovascular collapse [17].

The treatment of a ruptured SAA necessitates knowledge and an aggressive surgical approach. There are no well-established guidelines because this is a rare condition. Regardless of symptoms or size, elective repair is preferred during pregnancy, while planning for pregnancy, and in liver transplant candidates. Regardless of the size, intervention is recommended in symptomatic aneurysms. Other SAAs without symptoms can be effectively monitored with serial imaging [18], and all aneurysms larger than 2 cm should be treated, regardless of symptoms. The procedures described are aneurysmectomy with splenectomy or left splenopancreatectomy, ligation of the proximal and distal splenic arteries, and aneurysmectomy for the distal, mid, and proximal third SAAs, respectively [19, 20]. Although splenic preservation is desirable, it is difficult to achieve in an emergency setting with a ruptured SAA [20]. For pseudoaneurysms and unruptured true aneurysms, angiography and embolization have been described [19].

In general, the use of endovascular interventions in the form of coil embolization or stent grafting has its place in SAA repair, and in many centers, this is considered the primary approach [21, 22]. However, in the case of our patient, due to the instability of the patient and the lack of an endovascular setting in the center, open surgery was preferred. The choice of surgical method depends on the general approaches of the center, access to various blocking agents equipment, access to staff at all hours of the day and night and in emergencies, and the anatomical characteristics of the splenic artery, including the degree of tortuosity of the artery, and also access to the artery through devices available are all important factors in decision making.

The current case is even more intriguing because the patient had pemphigus vulgaris and was on steroid therapy. Although vessels are rarely involved in pemphigus vulgaris, rheumatologic disorders have been linked to vasculitis, and several cases have been reported in which aneurysms in the splenic artery have developed as a result of vasculitis involvement [23, 24]. Aydın *et al.* also reported the rupture of a splenic aneurysm in a male patient with sarcoidosis [25]. The most common cause of death in Behcet’s disease is an arterial aneurysmal rupture [26]. Akiyama *et al.* [27] also proposed that the long-term use of corticosteroids can weaken the arterial walls, and subsequently increase the risk of rupture. However, there is not enough evidence to support the increased risk of an aneurysm in rheumatological diseases. A spontaneous splenic artery dissection rupture may be diagnosed only in the presence of relevant symptoms, such as a limited retroperitoneal hematoma visible, for example, with a contrast-enhanced CT scan followed by a more specific technique, such as selected angiography, given the extreme rarity of this disease, and the one-of-a-kind case with successful treatment. Further reporting of such entities is required, along with a detailed pathophysiological evaluation to obtain the necessary information to draw a clear conclusion.

## Conclusion

Aneurysms of the splenic artery are a rare type of aneurysm that are often asymptomatic. It is essential to consider splenic aneurysm rupture as a second-line differential diagnosis, as this can lead to timely and appropriate lifesaving intervention.

## Data Availability

All data regarding this study has been reported in the manuscript. Please contact the corresponding author if you are interested in any further information.

## Abbreviations

BUN: Blood urease nitrate
CT: Computed tomography
SAA: Splenic artery aneurysm
